# Chronic widespread pain is associated with worsening frailty in European men

**DOI:** 10.1093/ageing/afv170

**Published:** 2015-12-17

**Authors:** Katie Fredrika Wade, David M. Lee, John McBeth, Rathi Ravindrarajah, Evelien Gielen, Stephen R. Pye, Dirk Vanderschueren, Neil Pendleton, Joseph D. Finn, György Bartfai, Felipe F. Casanueva, Gianni Forti, Aleksander Giwercman, Ilpo T. Huhtaniemi, Krzysztof Kula, Margus Punab, Frederick C. W. Wu, Terence W. O'Neill

**Affiliations:** 1 Arthritis Research UK Centre for Epidemiology, Institute of Inflammation and Repair, Manchester Academic Health Science Centre, The University of Manchester, Manchester, UK; 2 NIHR Manchester Musculoskeletal Biomedical Research Unit, Central Manchester University Hospitals NHS Foundation Trust—Manchester Academic Health Science Centre, Manchester, UK; 3 Cathie Marsh Institute for Social Research, The University of Manchester, Manchester, UK; 4 Manchester Royal Infirmary, Central Manchester University Hospitals NHS Foundation Trust, Manchester, UK; 5 Geriatric Medicine Department, Katholieke Universiteit Leuven, Leuven, Belgium; 6 Laboratory of Clinical and Experimental Endocrinology, Catholic University of Leuven, Leuven, Belgium; 7 Institute of Brain, Behaviour and Mental Health, The University of Manchester, Salford, UK; 8 Department of Obstetrics, Gynaecology and Andrology, Albert Szent-György Medical University, Szeged, Hungary; 9 Department of Medicine, Santiago de Compostela University, Santiago de Compostela, Spain; 10 Department of Experimental Clinical and Biomedical Sciences, University of Florence, Florence, Italy; 11 University of Lund—Malmö University Hospital, Lund, Sweden; 12 Department of Surgery and Cancer, Imperial College London, London, UK; 13 Department of Andrology and Reproductive Endocrinology, Medical University of Łódź, Łódź, Poland; 14 Andrology Unit, United Laboratories of Tartu University Clinics, Tartu, Estonia; 15 Andrology Research Unit, Developmental and Regenerative Biomedicine Research Group, Manchester Academic Health Science Centre (MAHSC), The University of Manchester, Manchester, UK

**Keywords:** frailty, pain, ageing, male health, EMAS

## Abstract

**Background:** we hypothesised that chronic widespread pain (CWP), by acting as a potential stressor, may predispose to the development of, or worsening, frailty.

**Setting:** longitudinal analysis within the European Male Ageing Study (EMAS).

**Participants:** a total of 2,736 community-dwelling men aged 40–79.

**Methods:** subjects completed a pain questionnaire and shaded a manikin, with the presence of CWP defined using the American College of Rheumatology criteria. Physical activity, smoking, alcohol consumption and depression were measured. Repeat assessments took place a median of 4.3 years later. A frailty index (FI) was used, with frail defined as an FI >0.35. The association between CWP at baseline and the new occurrence of frailty was examined using logistic regression; the association between CWP at baseline and change in FI was examined using negative binomial regression.

**Results:** at baseline, 218 (8.3%) men reported CWP. Of the 2,631 men who were defined as non-frail at baseline, 112 (4.3%) were frail at follow-up; their mean FI was 0.12 (SD 0.1) at baseline and 0.15 (SD 0.1) at follow-up, with a mean change of 0.03 (SD 0.08) *P* ≤ 0.001. Among men who were non-frail at baseline, those with CWP were significantly more likely to develop frailty. After adjustment for age and centre, compared with those with no pain, those with CWP at baseline had a 70% higher FI at follow-up; these associations remained significant after further adjustment for smoking, body mass index, depression, physical activity and FI at baseline.

**Conclusion:** the presence of CWP is associated with an increased risk of frailty in older European men.

## Introduction

Frailty is a common geriatric syndrome that becomes more prevalent with increasing age. Data pooled from 21 studies indicate an overall prevalence estimate of 10.7% in community-dwelling older adults [1]. There is no generally accepted definition of frailty; however, it has been defined as a ‘state of increased vulnerability to poor resolution of homeostasis after a stressor event, resulting from cumulative loss of physiological reserve’ [2]. Those suffering from frailty have been found to have an increased risk of hospitalisation and falls [3]. Factors linked with the development and progression of frailty include smoking, alcohol consumption and depression [4], obesity [5] and the presence of chronic diseases such as cardiovascular disease and diabetes [6]. Chronic widespread pain (CWP) is a prominent symptom of fibromyalgia, affecting up to 11% of adult men and women [7] and it is linked with adverse health outcomes including an increased mortality risk [8]. In this study, the American College of Rheumatology (ACR) criteria were used to identify those suffering from CWP [9]; subjects had to have pain for a minimum of 3 months in both the left and right sides of the body, above and below the waist and in the axial skeleton. CWP appears to be fundamentally different in its aetiology compared with more localised rheumatic or osteoarthritic pains. Risk factors for CWP include psychological distress [10], poor sleep [11], low levels of physical activity and being overweight [12, 13]. Perturbations in the Hypothalamic–Pituitary–Adrenal axis have been linked with the development of chronic pain [14], and we hypothesised that CWP, by acting as a stressor, would be associated with an increased risk of incident frailty. Previous studies provide support for a link between pain and frailty, although they have been unable to determine the direction of the observed associations because of the cross-sectional nature of the data [15, 16].

The aim of this analysis was to determine whether the occurrence of CWP predisposes middle-aged and older men to the development of, or worsening, frailty.

## Methods

### Study design

Details regarding recruitment and study set-up have been described elsewhere [17]. Briefly, 3,369 community-dwelling men aged 40–79 were recruited from population registers in eight centres: Florence (Italy), Leuven (Belgium), Łódź (Poland), Malmö (Sweden), Manchester (UK), Santiago de Compostela (Spain), Szeged (Hungary) and Tartu (Estonia). Subjects were mailed a study information pack, a short postal questionnaire, and they attended a health screening at a local research clinic. Of those who completed baseline assessments, 86% (adjusted for mortality) completed the follow-up assessments with a mean of 4.3 years (range 3.0–5.7 years; SD = 0.3) after baseline data were collected [18]. All assessments were carried out using standardised protocols. Ethical approval for the study was obtained in accordance with local institutional requirements in each centre. All subjects provided written informed consent.

### Assessments

#### Postal questionnaire

The initial postal questionnaire captured demographic, health and lifestyle information including smoking and alcohol consumption. Subjects were also asked whether they were currently being treated for asthma, chronic bronchitis, adrenal and/or prostate disease, epilepsy, peptic ulcer, thyroid disease, heart, kidney and/or liver conditions, testicular and/or pituitary disease, high blood pressure, diabetes, and if they had ever suffered a stroke or been diagnosed with cancer.

#### Other assessments

Subjects who attended for assessment completed an interviewer-assisted questionnaire that included the Beck's Depression Inventory-II (BDI-II) [19], the 36-Item Medical Outcomes Study Survey (SF-36) [20] and the Physical Activity Scale for the Elderly (PASE) [21]. Physical function tests included the Reuben's Physical Performance Test (PPT) [22] and Tinetti's balance and postural stability index [23]. Three neuropsychological tests were conducted to evaluate cognitive function; these measured visuo-perceptual abilities, executive function and memory [24], the recognition component of visual memory retrieval [25] and cognitive processing speed and visual scanning [26]. These assessments were used to provide deficits to make up the FI (Supplementary data, Appendix 1, available in *Age and Ageing* online). Anthropometric measurements included height, weight, mid to upper arm circumference and triceps skin fold thickness. Body mass index (BMI) was calculated as body weight (kg) divided by the square of height (m^2^).

### Frailty

An EMAS FI was developed comprising 39 deficits that represent symptoms, signs or functional impairments that accumulate with age and are individually related to adverse health outcomes [27]. The variables used fulfilled the requirements for the development of an FI; they related to health status, increased with age, did not appear too early in life and covered a range of systems. The deficits included co-morbidities and cognitive functioning, as well as items from the BDI-II, the SF-36, the PTT and the Tinetti index (Supplementary data, Appendix 1 available in *Age and Ageing* online). The same deficits were used in the FI at baseline and follow-up. Binary variables were re-coded; ‘0’ represented the absence of and ‘1’ the presence of a deficit. A value of ‘0.5’ was used for categorical variables that indicated an intermediate response. Continuous variables were dichotomised based on the 10th centile. The FI was calculated as the number of health deficits present in an individual divided by the number of deficits considered. The FI was categorised using published criteria [28]; robust subjects had FI scores <0.2, pre-frail subjects were 0.2–0.35 and frail subjects were >0.35. The EMAS FI has been associated with an increased risk of mortality [29] and impaired overall sexual functioning [30].

### Ascertainment of pain status

Subjects were asked if they had experienced ‘any pain which lasted for one day or more’ in the past month. Those who answered positively were then asked if their pain lasted 3 months or more and to indicate by shading a pain manikin. Those satisfying the ACR criteria for CWP were identified. Those participants who did report pain, but did not meet these criteria, were classified as experiencing ‘some pain’ and those who did not report any pain were classified as experiencing ‘no pain’. Using this definition in EMAS, CWP has previously been associated with poor mental health, the presence of co-morbidities and recent adverse life events [31].

### Statistical methods

Analyses were conducted using STATA SE v13.1 (StataCorp, College Station, TX, USA). Depending on distribution, parametric (paired *t*-tests) and non-parametric (Wilcoxon rank sum) statistical approaches were used to describe differences in baseline characteristics between those non-frail at baseline and those who did or did not develop frailty at follow-up. Logistic regression was used to determine the association between CWP at baseline and the new occurrence of frailty. To facilitate the longitudinal analysis, only subjects who were either robust or pre-frail at baseline were included in these models. Analyses were performed unadjusted, then adjusted for baseline age and centre and subsequently for baseline BMI, depression, smoking status and physical activity. The results were expressed as odds ratios (OR) and 95% confidence intervals (CI). Negative binomial regression was used to determine the association between CWP at baseline and an increase in FI at follow-up, with the change in FI as the dependent variable and CWP the main predictor. Negative binomial regression is typically used to model over-dispersed count variables. To facilitate this modelling approach, the FI was first multiplied by 100 to convert it to a scale from 0 to 100 (to yield integer values without changing the distribution), with 0 representing no deficits and 100 maximum deficits. Analyses were performed unadjusted, then adjusted for baseline age and centre, and subsequently for baseline BMI, depression, smoking status, physical activity and FI. As two items of the BDI-II were included as deficits in the FI; assessing changes in sleeping patterns and concentration; these were removed from the BDI-II score before running the analysis in order to avoid spurious associations being found. The results were reported as incident rate ratios (IRR) and 95% CIs.

## Results

### Subjects

Of the 3,369 men who took part in EMAS, 2,736 subjects, mean age 59.2 years (SD 10.6), had complete data to allow FI characterisation at baseline and follow-up. The baseline characteristics for these subjects are presented in Table 1. The mean BMI was 27.7 kg/m^2^ (SD 4.0), 23.3% drank alcohol on 5 or more days per week and 19.8% were current smokers.

**Table 1.** Subject characteristics

Baseline variables	All		Robust or pre-frail, both time points		Non-frail at baseline, frail at follow-up		*P* value*
	*n*	Mean (SD)	*n*	Mean (SD)	*n*	Mean (SD)	
Age (years)	2,736	59.2 (10.6)	2,519	58.4 (10.4)	112	67.4 (9.3)	≤0.001
Height (m)	2,712	174.0 (7.2)	2,499	174.3 (7.2)	112	171.1 (7.3)	≤0.001
Weight (kg)	2,706	83.9 (13.7)	2,492	83.6 (13.4)	112	86.2 (13.6)	0.02
Body mass index (kg/m^2^)	2,703	27.7 (4.0)	2,491	27.5 (3.9)	112	29.4 (3.9)	≤0.001
Physical activity scale for the elderly	2,540	200.1 (88.9)	2,338	205.1 (87.1)	102	156.4 (87.4)	≤0.001
Becks depression inventory (BDI-II)	2,736	6.5 (6.2)	2,519	5.9 (5.5)	112	11.0 (6.6)	≤0.001
BDI minus items used in frailty index	2,735	5.4 (5.5)	2,519	4.8 (4.9)	112	9.3 (6.1)	≤0.001
		Number (%)		Number (%)		Number (%)	
Alcohol intake (≥5 days/week)	2,725	634 (23.3)	2,509	615 (24.5)	112	12 (10.7)	≤0.001
Smoking status (current)	2,712	537 (19.8)	2,496	487 (19.5)	112	29 (25.9)	0.10
Pain status	2,624		2,419		106		≤0.001
Pain free		1,094 (41.7)		1,051 (43.4)		30 (28.3)	
Some pain		1,312 (50.0)		1,200 (49.6)		53 (50.0)	
Chronic widespread pain		218 (8.3)		168 (6.9)		23 (21.7)	

*Paired *t*-test or Wilcoxon signed-rank test depending on variables distribution; *χ*^2^ test for categorical variables.

### Change in frailty status

At baseline, 2,244 men were classified as robust, 387 men as pre-frail and 105 as frail and at follow-up; 2,079 robust, 478 pre-frail and 179 frail. Of those, 2,631 men were non-frail at baseline, 112 (4.3%) became frail at follow-up. The mean score for the FI at baseline was 0.12 (SD 0.1) and at follow-up was 0.15 (SD 0.1); the mean change was 0.03 (SD 0.08). The distribution of the FI at baseline and follow-up is shown in Supplementary data, Appendix 2, available in *Age and Ageing* online. Complete description of frailty status by baseline pain is shown in Supplementary data, Appendix 3, available in *Age and Ageing* online.

### Determinants of incident frailty

Among the 2,631 men non-frail at baseline, compared with those who did not develop frailty at follow-up, those who did were older, had lower physical activity levels, were more likely to be depressed, had a higher BMI and were more likely to report experiencing CWP at baseline (21.7% versus 6.9%, *P* ≤ 0.001), see Table 1.

### Pain status and frailty

#### Incident frailty

Table 2 shows the associations between baseline pain and incident frailty at follow-up. After adjusting for age and centre, compared with subjects who reported no pain, those who reported some pain at baseline were more likely to have developed frailty at follow-up (OR = 1.59; 95% CI 1.00, 2.55). Compared with those without pain, those with CWP had an increased risk of frailty at follow-up (OR = 5.14; 95% CI 2.82, 9.38). Further adjustment for baseline BMI, depression, smoking status and physical activity attenuated the results; compared with those with no pain, those experiencing CWP at baseline were significantly more likely to have developed frailty at follow-up (OR = 4.32; 95% CI 2.21, 8.46).

**Table 2.** Baseline pain status and incident frailty: logistic regression analysis

Baseline characteristics	Model 1	Model 2	Model 3
	Odds ratios (95% confidence intervals)		
Age (years)		1.10 (1.07, 1.12)**	1.10 (1.07, 1.13)**
Pain status			
No pain	Reference	Reference	Reference
Some pain	1.55 (0.98, 2.44)	1.59 (1.00, 2.55)*	1.46 (0.87, 2.45)
Chronic widespread pain	4.80 (2.72, 8.46)**	5.14 (2.82, 9.38)**	4.32 (2.21, 8.46)**
Body mass index (BMI) (kg/m^2^)			1.10 (1.04, 1.16)**
Depression (BDI)			1.11 (1.08, 1.15)**
Smoking (yes versus no)			2.52 (1.44, 4.42)**
Physical activity scale for the elderly			0.99 (0.98, 0.99)*

Model 1: unadjusted, *n* = 2,525; Model 2: adjusted for age and centre, *n* = 2,525; Model 3: adjusted for age, centre, BMI, smoking status, depression (continuous variable minus items used in FI) and physical activity at baseline, *n* = 2,479.

*P ≤ 0.05.

**P ≤ 0.001.

#### Worsening frailty

After adjusting for age and centre, compared with subjects who reported no pain, those who reported CWP at baseline had an estimated 70% higher FI score at follow-up, and those with some pain had a 30% higher FI score at follow-up. After further adjustment for baseline BMI, depression, and smoking status, physical activity and FI, the strength of the association was attenuated though remained significant. Compared with subjects who reported no pain, those who reported some pain, had an estimated 10%, and those who reported CWP had an estimated 18% higher FI score at follow-up, see Table 3. As there is a reported strong association between CWP and sleep [10, 11], the data were re-analysed after excluding an item of the BDI-II that related to changes in sleeping patterns, which was used in the derivation of the FI; CWP remained predictive after adjusting for age and centre (IRR = 1.68; 95% CI 1.51, 1.87) and after adjusting further for baseline BMI, depression, smoking, physical activity and baseline FI (IRR = 1.18; 95% CI 1.07, 1.30).

**Table 3.** Baseline pain status and change in frailty index: negative binomial regression analysis

Baseline characteristics	Model 1	Model 2	Model 3
	Incident rate ratios (95% confidence intervals)		
Age (years)		1.04 (1.03, 1.04)**	1.02 (1.02, 1.02)**
Pain status			
No pain	Reference	Reference	Reference
Some pain	1.30 (1.22, 1.40)**	1.30 (1.22, 1.38)**	1.10 (1.04, 1.15)**
Chronic wisespread pain	1.75 (1.55, 1.98)**	1.70 (1.52, 1.88)**	1.18 (1.08, 1.30)**
Body mass index (BMI) (kg/m^2^)			1.02 (1.01, 1.02)**
Depression (BDI-II)			1.02 (1.01, 1.02)**
Smoking (yes versus no)			1.18 (1.11, 1.26)**
Physical activity scale for the elderly			0.99 (0.99, 1.00)
Frailty index (baseline)			1.04 (1.03, 1.04)**

Model 1: unadjusted, *n* = 2,624; Model 2: adjusted for age and centre, *n* = 2,624; Model 3: adjusted for age, centre, BMI, smoking status, depression (continuous variable minus items used in FI), physical activity and FI at baseline, *n* = 2,572.

**P ≤ 0.001.

## Discussion

In this prospective study, CWP was associated with the development of, and also worsening, frailty as assessed using an FI. This was independent of previously identified risk factors such as smoking status, BMI and depressive symptoms.

Our data are consistent with the findings from earlier studies [15, 16]. In a cross-sectional study of 1,705 men aged 70 or older, an association was found between frailty, defined using the Cardiovascular Health Study criteria, and intrusive pain (determined using a question from the Short-form 12-item health survey: ‘During the past four weeks, how much did pain interfere with your normal work—including both work outside the home and housework?’), after adjustment for demographic factors, number of co-morbidities and arthritis [15]. In a cross-sectional study of 4,968 men and women, moderate or greater levels of pain (assessed using a 5-point verbal descriptor scale: ‘How much bodily pain have you had during the past 4 weeks?’) were found to be associated with frailty, measured using self-report items from the Canadian Study of Health and Ageing [16]. A key limitation with these cross-sectional studies is their inability to determine the temporal nature of the association. Our longitudinal data confirm an association between a baseline assessment of CWP and the subsequent development of, and worsening of, frailty over 4.3 years of follow-up.

We can hypothesise possible mechanisms linking chronic pain with the development of frailty. Chronic pain is associated with a variety of adverse health factors including depression and comorbidities. Karp *et al.* [32] argued that, due to the multidimensional impact CWP has on older people, it is plausible that it impacts on physiologic systems, reduces reserve and decreases one's ability to maintain homeostasis. This view was supported by Shega *et al.* [16], who argued that older adults with persistent pain and its associated conditions, including lack of sleep and poor nutrition, may experience a decrease in their reserve, which increases the likelihood of falls and cognitive dysfunction. The evidence that perturbations in the stress-hormone axis are linked with the development of CWP would be consistent with this [14]. The strength of the association found between pain and frailty in the current study would appear to be stronger for CWP than some pain. This may be explained by CWP having more profound effects on a number of health outcomes than regional/short-lived pain [33]. CWP is also recognised by ACR as a meaningful clinical capture of severe pain, whereas it is not clear what the measure for ‘some pain’ is capturing, which could suggest that there is a dose–response relationship between pain and frailty.

Our findings are also consistent with earlier studies reporting associations between frailty and a variety of health and lifestyle factors. Hubbard and colleagues [34] reported that heavy smokers (determined through self-report) were the most frail (using an FI) compared with light and never smokers. Blaum and colleagues [5] conducted a cross-sectional analysis on 599 women aged 70–79 and found that obesity (BMI ≥30 kg/m^2^) was associated with frailty (defined using a ‘frailty phenotype’ approach). Strawbridge and colleagues [4] carried out analyses on prospective risk factors that were measured in 574 subjects aged 65–102, over three decades. Frailty was categorised based on whether subjects had difficulties in two or more functional domains; both smoking and depression were cumulative predictors of frailty. Past research has also identified an association between frailty and social deprivation [35] and future research should take this into consideration when exploring the association between pain and frailty.

Our study has unique positive features; the prospective data were derived from a large population-based sample; a standardised approach was taken to the conduct of the study and established and validated measures were used to capture CWP and frailty. There are limitations; the response rate for participation at baseline was 41% with those who declined to take part being older, more likely to be current smokers and reporting experiencing less pain lasting at least 1 day in the past month, than those who participated in the study [17]. Accordingly, the estimates of the occurrence of both frailty and CWP at baseline may differ from the base populations from which the subjects were recruited, and caution is required in interpreting these absolute estimates. Similarly, a number of men did not attend for the repeat survey; some had died while others declined to participate. Given this, the results relating to the absolute incidence of frailty need to be interpreted with some caution. It seems unlikely that any such selection factors would impact on the findings reported here which were based on internal comparison of those who took part. The data on CWP in this study were obtained by self-report and a shaded manikin—it is possible that errors of recall or shading in the manikin may have resulted in some misclassification of exposure status (CWP); the effect of any such misclassification though would be to attenuate any real biological association. Finally, the cohort studied comprised predominantly Caucasian men, with a mean age of 59 years; therefore, caution is needed when extrapolating the findings beyond this group

In conclusion, CWP is associated with both incident, and worsening frailty in middle-aged and older European men, largely independent of health and lifestyle confounders. Our findings not only suggest potential opportunities for targeted interventions to reduce the occurrence of frailty, but also highlight that clinicians need to be more attentive of the pain status of older people. Further studies are needed to confirm our observations and identify the physiological mechanisms underpinning this association.

**Key points**

- CWP was associated with worsening frailty among older men, as assessed using an FI.
- This association was largely independent of various health and lifestyle factors.
- The aetiology of the relationship between pain and frailty requires further study.

## Conflicts of interest

None declared.

## Supplementary data

Supplementary data mentioned in the text are available to subscribers in *Age and Ageing* online.

Supplementary Data
