# Supplementary material for: Chronic widespread pain is associated with worsening frailty in European men
Source: Age Ageing. 2015 Dec 17;45(2):268–74. doi: 10.1093/ageing/afv170 (PMC4776622; doi:10.1093/ageing/afv170)
Supplement: Supplementary Data [file supp_45_2_268__index.html]

Chronic widespread pain is associated with worsening frailty in European men — Supplementary Data 

# Chronic widespread pain is associated with worsening frailty in European men

## Supplementary Data

Supplementary Data

- Supplementary Data - Doc file
