# Supplementary material for: Chronic widespread pain is associated with worsening frailty in European men
Source: Age Ageing. 2015 Dec 17;45(2):268–74. doi: 10.1093/ageing/afv170 (PMC4776622; doi:10.1093/ageing/afv170)
Supplement: Supplementary Data [file supp_afv170_afv170supp.doc]

**SUPPLEMENTARY MATERIAL**

**Appendix 1** Health deficit variables included in the EMAS-FI

| Origin | Variable |
| --- | --- |
| SF36 | Rating general health |
| SF36- Activity Daily Living | Help Feeding yourself |
|  | Help Walking in your home |
|  | Help Bathing and dressing yourself |
|  | Walking 1 km |
|  | Walking more than 1 km |
|  | Climbing one flight of stairs |
|  | Climbing several flights of stairs |
|  | Unable to do moderate activity |
|  | Unable to do vigorous activity |
| During the past 4 weeks have you had any of the following problems |  |
| SF36 | Accomplish less than you would like as a result of your physical health |
| SF36 | Cut down on the amount of time spent on work or other activities as a result of emotional problems |
| Questions are about how you feel and how things have been with you during the past 4 weeks |  |
| SF36 | Full of life |
|  | In the dumps |
|  | Down hearted |
|  | Tired |
| SF36-During the last six months have you experienced | Serious illness or injury to yourself |
| Beck depression inventory BDI | Change in sleep pattern |
|  | Concentration |
| International Prostate Symptom Score | Over the past month, how often have you had to |
|  | Postpone urination |
|  | Night urinate |
|  | Weak Stream |
| Self reported morbidities |  |
|  | Heart condition |
|  | High blood pressure |
|  | Bronchitis |
|  | Asthma |
|  | Diabetes |
|  | Liver condition |
|  | Kidney condition |
|  | Prostate disorder |
|  | Thyroid disorder |
|  | Cancer ever |
|  | Stroke ever |
|  |  |
| Cognition | Copying -Rey-Osterrieth Complex Figure (ROCF) |
|  | Delayed reproduction- Rey-Osterrieth Complex Figure (ROCF) |
|  | Camden Topographical Recognition Memory (CTRM) |
|  | Digit-Symbol Substitution (DSST) test |
| Tinetti |  |
|  | Tinetti total |
| Physical performance test | Time to walk 15.4 meters |

**Appendix 2** The distribution of the FI at baseline and follow-up


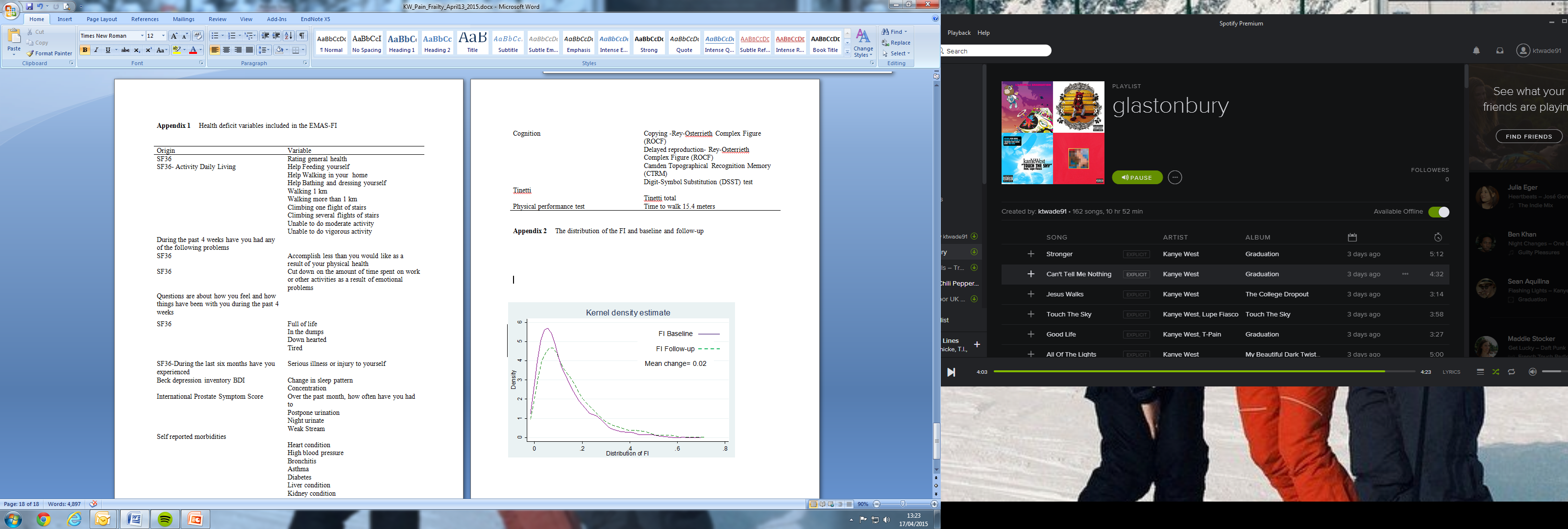


**Appendix 3** Frailty status at baseline and follow-up: by baseline pain status

|  | **Baseline Pain** | | |
| --- | --- | --- | --- |
|  | **No pain** | **Some pain** | **Chronic Widespread Pain** |
| **Baseline Frailty** | **N (%)** | **N (%)** | **N (%)** |
| **Robust** | 978 (89.4) | 1035 (79.0) | 146 (67.0) |
| **Pre-frail** | 103 (9.4) | 218 (16.6) | 45 (20.6) |
| **Frail** | 13 (1.2) | 59 (4.5) | 27 (12.4) |
| **Follow-up Frailty** |  |  |  |
| **Robust** | 912 (83.4) | 969 (73.9) | 127 (58.3) |
| **Pre-frail** | 143 (13.1) | 255 (19.4) | 51 (23.4) |
| **Frail** | 1. (3.6) | 88 (6.7) | 40 (18.4) |
